# Effect of Surface Treatments on Repair Bond Strength of Aged Bulk-Fill Resin Composites

**DOI:** 10.3390/polym17172326

**Published:** 2025-08-28

**Authors:** Mashael Binhasan, Faisal Althobaiti, Rakan Alyami, Khalid Aljabri, Talal Alabbas, Haifa Barakah

**Affiliations:** 1 Department of Restorative Dentistry, College of Dentistry, King Saud University, Riyadh 11451, Saudi Arabia; 2 Intern, College of Dentistry, King Saud University, Riyadh 11451, Saudi Arabia

**Keywords:** restorative dentistry, resin composite repair, bond strength testing, aging of dental composites

## Abstract

This study evaluated the influence of different surface treatments and composite materials on the microtensile bond strength (μTBS) of repaired aged bulk-fill resin composite restorations, aligning with the principles of minimal intervention dentistry. Seventy-two specimens of bulk-fill resin composite (Tetric EvoCeram) were prepared, sectioned into bars (1 × 1 × 5 mm), and subjected to thermocycling to simulate aging. Specimens were randomly allocated into six groups (n = 12) based on surface treatment and repair material: phosphoric acid etching followed by repair with either Tetric EvoCeram (Group 1) or Filtek Z350 XT (Group 2); diamond bur roughening followed by repair with Tetric EvoCeram (Group 3) or Filtek Z350 XT (Group 4); and air abrasion followed by repair with Tetric EvoCeram (Group 5) or Filtek Z350 XT (Group 6). μTBS testing was performed using a universal testing machine, and failure patterns were examined under a stereomicroscope at 40× magnification. The highest bond strength values were observed in Groups 5 and 6 (air abrasion), followed by Group 3 (diamond bur). Groups 1, 2, and 4 showed significantly lower bond strength values (*p* < 0.05). No significant differences in failure modes were reported across groups. These findings suggest that air abrasion is a superior surface treatment for repairing aged bulk-fill resin composites, as it significantly enhanced μTBS compared to phosphoric acid etching and diamond bur roughening.

## 1. Introduction

Composite restorations were first introduced to the dental market in the 1960s and have since gained widespread popularity. Today, they are the most commonly used materials for direct anterior and posterior restorations due to their esthetic characteristics, strong adhesion to tooth structure, lower cost compared to indirect restorations, and favorable clinical performance with minimal annual failure rates [1]. However, wear, fractures, and marginal defects are common reasons for restoration failure. Data suggest that complete removal and replacement of restorations carry a biological cost, often requiring enlargement of the cavity and loss of additional sound tooth structure, which in turn weakens the tooth and may lead to pulpal inflammation [1,2,3]. The concept of minimal intervention dentistry promotes repair over replacement whenever feasible. Repair refers to selectively replacing the defective portion of a restoration with fresh composite, preserving the sound segments [1,4]. In addition to being conservative, repair is associated with shorter chairside time, better preservation of tooth structure, and extended longevity of both the restoration and the tooth. Thus, repair should be considered the first-line option in managing defective composite restorations [1,5]. Bulk-fill (BF) resin composites have been developed to simplify restorative procedures by enabling placement in thicker increments. However, the literature indicates variability in their properties. A systematic review of 13 studies, covering both high- and low-viscosity BF composites, reported considerable heterogeneity in composition and translucency, which might influence clinical outcomes [6].

After extended clinical service, composite restorations often require repair rather than full replacement. Therefore, evaluating the factors that influence the success of composite repair, especially the bond strength between aged and new materials, is crucial. This challenge arises from the variability of the bonding substrate. In many clinical scenarios, the original composite’s manufacturer or batch number was not recorded, making it difficult to replicate exact repair protocols. Additionally, the dental market offers a wide variety of composite resins with differing compositions and structures, further complicating consistency in repair outcomes [7,8]. Beyond these material-based inconsistencies, several other factors can significantly impact repair bond strength. These include the matrix composition and filler characteristics (such as load, size, and distribution) of the composite, as well as the surface treatment method used to prepare the aged restoration and the degree of micromechanical retention achieved [8,9,10]. Although several studies have investigated repair strategies for conventional resin composites, there is limited evidence on the optimal surface treatment methods for aged bulk-fill resin composites, particularly in relation to different repair materials. This gap in the literature restricts clinical guidance on minimally invasive repair protocols. The aim of the current study was to assess the influence of different surface treatment techniques and composite materials on the microtensile bond strength (μTBS) of repaired aged bulk-fill resin composites. Understanding these variables is critical for optimizing clinical repair strategies and improving long-term dental restoration outcomes. It was hypothesized that air abrasion would result in superior bond strength compared to phosphoric acid etching and diamond bur roughening, regardless of the repair composite used.

## 2. Materials and Methods

### 2.1. Specimen Preparation

A total of 72 specimens were prepared (n = 12 per group). Nanohybrid bulk-fill resin composite (Tetric EvoCeram Bulk Fill, Ivoclar Vivadent, Liechtenstein, Austria) was packed into prefabricated metal molds (12 mm × 12 mm × 7 mm) placed on a glass plate. The material was condensed using a plastic instrument, and another glass plate was used to achieve a flat surface. The composite was light-cured for 20 s using an LED curing unit (Bluephase G2, Ivoclar Vivadent, Austria), following the manufacturer’s instructions. Cured blocks were then sectioned into bar-shaped specimens (1 mm × 1 mm × 5 mm) using a precision cutter (IsoMet™ Low-Speed Precision Cutter, Buehler, IL, USA).

### 2.2. Aging and Repairing Procedures

All specimens underwent artificial aging through thermocycling (5000 cycles) between 5 °C and 55 °C, with a dwell time of 30 s and a transfer time of 15 s per cycle. After surface treatment and repair, specimens underwent an additional 5000 thermocycles using the same protocol to simulate intraoral aging [11].

Two composite materials were used to repair the aged specimens: nanohybrid bulk-fill resin composite (Tetric EvoCeram Bulk Fill, Ivoclar Vivadent, Liechtenstein, Austria) and nanofilled resin composite (Filtek Z350 XT, 3M ESPE, St. Paul, MN, USA). Table 1 summarizes the characteristics of the materials used.

### 2.3. Grouping of Specimens

The 72 specimens were randomly divided into six groups (n = 12 each) according to the surface treatment method and the type of repair composite used. Groups 1 and 2 received phosphoric acid etching followed by repair with Tetric EvoCeram and Filtek Z350 XT, respectively. Groups 3 and 4 were subjected to diamond bur roughening, with Tetric EvoCeram used in Group 3 and Filtek Z350 XT in Group 4. Groups 5 and 6 underwent air abrasion treatment, followed by repair with Tetric EvoCeram and Filtek Z350 XT, respectively.

### 2.4. Surface Treatments and Composite Application

Each specimen was secured in a silicone mold and subjected to surface treatments according to group assignment. In Groups 1 and 2, surfaces were etched with 37% phosphoric acid for 15 s, rinsed, dried, and treated with Tetric N-Bond Universal adhesive, which was air-thinned and light-cured for 20 s. Adhesive air thinning was standardized by applying a gentle stream of compressed air from a distance of approximately 10 cm for 5 s, following the manufacturer’s instructions. In Groups 3 and 4, surface roughening was performed using a coarse-grit diamond bur (No. 027, Brasseler, Savannah, GA, USA) for 10 s under water cooling by a single operator to ensure consistent pressure; burs were replaced every five specimens. The adhesive protocol was then applied. In Groups 5 and 6, surface treatment was performed using an intraoral air abrasion unit (Danville, CA, USA) at 120 psi for 30 s, applied perpendicularly to the surface from a 5 mm distance, followed by the same adhesive procedure. Air abrasion was performed using 50 µm alumina particles under standardized conditions. After surface treatments, the corresponding repair composite (Tetric EvoCeram or Filtek Z350 XT) was packed onto the treated surface using the silicone mold to form a bar measuring 1 mm × 1 mm × 10 mm.

### 2.5. μTBS Testing and Failure Mode Analysis

μTBS testing was conducted using a universal testing machine (Instron, Norwood, MA, USA) at a crosshead speed of 1 mm/min. Bars were individually fixed using cyanoacrylate glue in a testing jig. Testing was performed at room temperature. Following fracture, the failure mode of each specimen was examined under a stereomicroscope at 40× magnification in a blinded manner and classified as adhesive (at the bonding interface), cohesive (within the composite), or mixed (a combination of both).

### 2.6. Statistical Analysis

Data were analyzed using SPSS software (Version 26.0, IBM Inc., Chicago, IL, USA). Descriptive statistics were calculated. Two-way ANOVA followed by Tukey’s post hoc test was used to compare μTBS values. Independent samples *t*-tests were used for specific comparisons. The chi-square test was applied for failure mode analysis. Statistical significance was set at *p* ≤ 0.05.

## 3. Results and Discussion

The findings revealed significant main effects for both surface treatment (*p* < 0.0001) and composite material type (*p* < 0.0001), while the interaction between them was not significant (*p* = 0.663) (Table 2). This indicates that both factors independently influenced the μTBS, but their combined interaction did not significantly affect the outcome.

**Figure 1:**
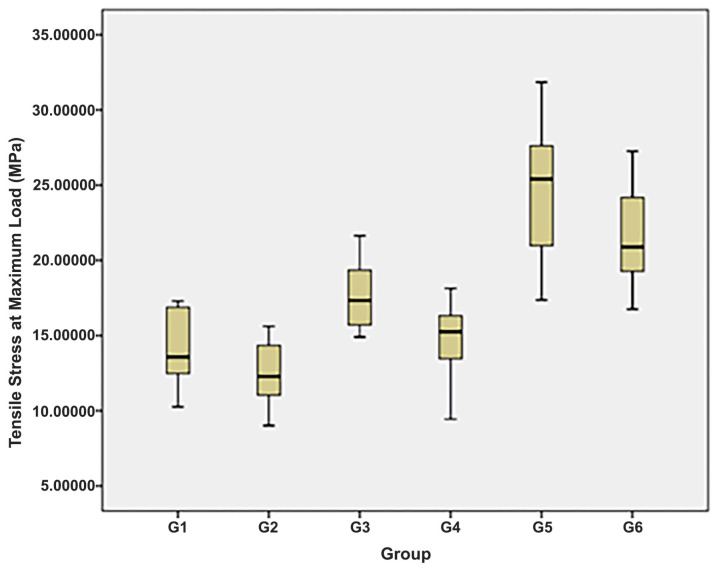
Mean values of μTBS across six study groups.

Analysis of μTBS values across the six experimental groups demonstrated significant variation (*p* < 0.0001; Table 3). Post hoc pairwise comparisons (Table 4) showed that Groups 1, 2, and 4 had the lowest mean μTBS values, with no significant difference among them (*p* > 0.05). Group 3 exhibited a significantly higher mean μTBS, while Groups 5 and 6 demonstrated the highest mean values, which were comparable and significantly greater than those of all other groups. These findings are visually summarized in Figure 1.

Independent *t*-tests comparing the μTBS between composites under the same surface treatments (Table 5) revealed no significant differences between Groups 1 and 2 (etched), nor between Groups 5 and 6 (air abraded). However, a significant difference was observed between Groups 3 and 4 (diamond bur), with Group 3 (same composite repair) showing higher bond strength (*p* = 0.007). Evidence from the literature indicates that the superior performance observed in same-composite repairs may be attributed to greater chemical affinity and monomer compatibility between the aged and repair materials, which can facilitate copolymerization at the interface and enhance bond strength compared with using a different composite [12].

Failure mode analysis is presented in Table 6. For specimens repaired with the same composite (Tetric EvoCeram), cohesive failure dominated across all surface treatments with no significant difference among groups (*p* = 0.793), while for specimens repaired with a different composite (Filtek Z350 XT), adhesive failures were more frequent, especially in the etched group, but differences were still not statistically significant (*p* = 0.455). These results indicated a predominance of cohesive failures when bulk-fill composites were repaired with the same material, whereas adhesive failures were more frequent when nanofilled composites were used for repair. Despite these trends, the chi-square test indicated no statistically significant differences in failure mode distribution across groups (*p* > 0.05).

These results support growing evidence in restorative dentistry that surface treatment significantly impacts the success of composite repair. In particular, these findings reinforce that air abrasion using aluminum oxide yields the most favorable bond strength outcomes. This can be attributed to the ability of air abrasion to create microretentive features and increase the surface area available for bonding, thereby enhancing adhesion. Moreover, air abrasion can effectively remove the outermost, degraded composite layer, exposing a fresher substrate more receptive to bonding. The superior μTBS observed in Groups 5 and 6 confirms the observations of previous studies reporting aluminum oxide air abrasion as a reliable method for composite repair, especially under aged conditions [9,12,13,14,15]. The superior performance of air abrasion compared to diamond bur roughening may be also explained by the more effective removal of the smear layer and the creation of a microretentive surface with increased surface energy, which enhances adhesive penetration and micromechanical interlocking.

In contrast, phosphoric acid etching alone resulted in the lowest bond strengths (Groups 1 and 2). This outcome suggests that etching may not sufficiently modify the aged composite surface to promote effective micromechanical interlocking. The literature supports that phosphoric acid functions primarily as a surface cleanser without significantly increasing surface roughness [9,16,17,18]. The reduced performance of phosphoric acid etching could also possibly be related to its limited ability to modify the surface energy and wettability of the aged composite, resulting in suboptimal micromechanical retention and poorer bonding effectiveness compared with air abrasion or diamond bur roughening.

Diamond bur roughening yielded intermediate results. Group 3 showed improved μTBS compared to acid etching, likely due to the increased surface area and removal of the superficial resin layer. However, Group 4 (diamond bur + nanofilled composite) had reduced bond strength, not significantly different from the acid-etched groups. This reduction may be due to the formation of a smear layer during roughening, which can obstruct adhesive infiltration and weaken bond integrity [19,20]. Reports remain mixed in views on the effectiveness of diamond burs for repair purposes, with some studies reporting no significant advantage [21], while others indicate improved outcomes [22,23]. The lower performance of diamond bur roughening with the nanofilled composite may be attributed to differences in filler size and distribution, polymer matrix composition, and degree of conversion, all of which can influence micromechanical retention and chemical bonding during repair. Differences in filler content and distribution between Tetric EvoCeram and Filtek Z350 XT may also contribute to the variation in repair bond strength, as filler characteristics can influence micromechanical interlocking potential, resin infiltration, and overall interfacial bonding.

In the current study, BF composites were selected because they are increasingly used in clinical practice due to their simplified placement in thicker increments, making it clinically relevant to investigate their repairability compared with conventional composites [7,8]. The material compatibility between the aged composite and the repair material may contribute to bond performance. When the same bulk-fill composite was used for repair, the bond was generally stronger than when a different composite was applied. The reason might be related to better chemical affinity and structural compatibility between identical materials. The observed trend of more cohesive failures in same-material groups further supports this suggestion, whereas the increased frequency of adhesive failures in cross-material repairs suggests weaker interfacial adhesion. These findings indicate that using the same composite material for repair may enhance bonding reliability.

The limitations of the current study include the in vitro setting. While maximum attempts were made for the in vitro setting to be standardized and controlled, it did not fully replicate the complexities of intraoral conditions, such as salivary enzymes, bacterial biofilms, and variable masticatory forces. Additionally, while thermocycling simulates thermal stress, it cannot encompass the full spectrum of oral degradation mechanisms. No power analysis was performed for the failure mode assessment, which may have reduced the ability to detect minimal group differences. Other limitations also include the absence of long-term hydrolytic degradation assessment, the absence of inter-operator reproducibility checks, and the narrow range of materials tested. Further research should explore longer-term aging protocols, alternative surface treatments (e.g., laser conditioning, silane coupling agents, and tribochemical silica coating), and material combinations to broaden applicability, as well as in vivo or in situ confirmation of in vitro findings.

## 4. Conclusions

This in vitro study demonstrated that the type of surface treatment significantly affects the repair bond strength of aged bulk-fill resin composites. Among the tested methods, aluminum oxide air abrasion yielded the highest μTBS, regardless of the composite type used for repair. Phosphoric acid etching alone resulted in the lowest bond strength, indicating limited effectiveness as a standalone surface treatment. Repairing with the same composite material provided better bond performance compared to using a different type, particularly under diamond bur roughening. Both the surface conditioning method and material compatibility play key roles in achieving durable composite repair.

The clinical significance of these findings lies in the fact that effective and durable repair of aged resin composite restorations can reduce the need for complete restoration replacement, minimizing treatment time and cost. This study highlights the clinical superiority of aluminum oxide air abrasion for enhancing repair bond strength and supports the use of the same restorative material whenever possible. These findings provide practical guidance for dental clinicians aiming to adopt minimally invasive strategies in restorative dental practice.

## Figures and Tables

**Table 1:** Materials used in this study.

Material	Manufacturer	Type	Shade	Filler Content and Composition
Filtek Z350 XT	3M ESPE, USA	Nanofilled	A2	82% wt (59.5% vol); Bis-GMA, UDMA, TEGDMA, PEGDMA, Bis-EMA; 20 nm nano silica, zirconia/silica agglomerates (5–20 nm), clusters (0.6–1.4 μm)
Tetric EvoCeram Bulk Fill	Ivoclar Vivadent, Liechtenstein	Bulk-fill nanohybrid	A2	76–77% wt (53–54% vol); Bis-GMA, UDMA, Ba-Al-Si-glass, prepolymer filler, mixed oxide
Phosphoric acid 37%	Scotchbond, 3M, Germany	Etchant	–	32% phosphoric acid (wt), pH ≈ 0.1
Tetric N-Bond Universal	Ivoclar Vivadent, Liechtenstein, Austria	Adhesive	–	Phosphoric acid acrylate, HEMA, Bis-GMA, UDMA, ethanol, initiators, stabilizers

**Table 2:** Two-way ANOVA results for μTBS.

Source	Type III Sum of Squares	df	Mean Square	*p*-Value
Surface treatment	1211.091	2	605.545	<0.0001 *
Composite type	121.293	1	121.293	<0.0001 *
Surface × composites	7.472	2	3.736	0.663

μTBS: microtensile bond strength; df: degrees of freedom. * Statistically significant at *p*-value < 0.05.

**Table 3:** Mean μTBS values (MPa) by group.

Group	Surface Treatment	Repair Material	Mean μTBS ± SD (MPa)	*p*-Value
G1	Phosphoric acid etching	Tetric EvoCeram	14.1943 ± 2.44	<0.0001 *
G2	Phosphoric acid etching	Filtek Z350 XT	12.5046 ± 2.13	
G3	Diamond bur roughening	Tetric EvoCeram	17.6419 ± 2.25	
G4	Diamond bur roughening	Filtek Z350 XT	14.6754 ± 2.65	
G5	Air abrasion	Tetric EvoCeram	24.6728 ± 4.49	
G6	Air abrasion	Filtek Z350 XT	21.5414 ± 3.39	

* Statistically significant at *p*-value < 0.05.

**Table 4:** Post hoc analysis of microtensile bond strength (μTBS) values across six study groups. Different superscript letters indicate statistically significant differences (*p* < 0.05).

Group	Surface Treatment + Repair Material	n	μTBS (MPa)
G2	Phosphoric acid + Filtek Z350 XT (FZ)	12	12.50 ^a^
G1	Phosphoric acid + Tetric EvoCeram (TEC)	12	14.19 ^a^
G4	Diamond bur + Filtek Z350 XT (FZ)	12	14.68 ^a^
G3	Diamond bur + Tetric EvoCeram (TEC)	12	17.64 ^b^
G6	Air abrasion + Filtek Z350 XT (FZ)	12	21.54 ^c^
G5	Air abrasion + Tetric EvoCeram (TEC)	12	24.67 ^c^

Groups listed in ascending order of their mean μTBS values. Groups sharing the same superscript letter are not significantly different.

**Table 5:** Comparison of mean values of μTBS between surface treatments for each composite material used.

Surface Treatment	Comparison	*p*-Value
Phosphoric acid etching	G1 (TEC) vs. G2 (FZ)	>0.05
Diamond bur roughening	G3 (TEC) vs. G4 (FZ)	0.007 *
Air abrasion	G5 (TEC) vs. G6 (FZ)	>0.05

TEC: Tetric EvoCeram; FZ: Filtek Z350 XT; μTBS: microtensile bond strength. * Statistically significant at *p*-value < 0.05.

**Table 6:** Distribution and comparison of failure modes across study groups.

Material Type	Group	Mode of Failure		χ^2^-Value	*p*-Value
		Adhesive, n (%)	Cohesive, n (%)		
Same Repair Material (Tetric EvoCeram)	Group 1: Etched surface	1 (8.33%)	11 (91.67%)		
	Group 3: Diamond bur roughening	2 (16.67%)	10 (83.33%)		
	Group 5: Air abrasion	2 (16.67%)	10 (83.33%)	0.465	0.793
Different Repair Material (Filtek Z350 XT)	Group 2: Etched surface	8 (66.67%)	4 (33.33%)		
	Group 4: Diamond bur roughening	7 (58.33%)	5 (41.67%)		
	Group 6: Air abrasion	5 (41.67%)	7 (58.33%)	1.575	0.455

χ^2^: chi-square test; n: number of specimens; %: percentage.

## Data Availability

The original contributions presented in this study are included in the article; further inquiries can be directed to the corresponding author.
